# HLA‐A ^∗^01:01 Allele Is Associated With Increased Risk of Transplant‐Related Mortality After MHC Matched Unmodified Allogeneic Hematopoietic Stem Cell Transplantation

**DOI:** 10.1155/jimr/7237488

**Published:** 2026-08-31

**Authors:** Emily A. Cowen, Samantha Brown, Rachel E. Reingold, Sarah Samorodnitsky, Maira Fonseca, Joseph R. Stoll, Sean M. Devlin, James W. Young, Samira A. Fatmi, Molly Maloy, Sergio Giralt, Doris M. Ponce, Ann A. Jakubowski, Alina Markova

**Affiliations:** ^1^ Dermatology Service, Department of Medicine, Memorial Sloan Kettering Cancer Center, New York, New York, USA, mskcc.org; ^2^ Department of Biostatistics and Epidemiology, Memorial Sloan Kettering Cancer Center, New York, New York, USA, mskcc.org; ^3^ Department of Dermatology, Weill Cornell Medical College, New York, New York, USA, cornell.edu; ^4^ Adult Bone Marrow Transplant Service, Department of Medicine, Memorial Sloan Kettering Cancer Center, New York, New York, USA, mskcc.org

**Keywords:** acute graft-versus-host-disease, allogeneic hematopoietic stem cell transplant, hematologic neoplasms, HLA-A1 antigen, human leukocyte antigen, transplant-related mortality

## Abstract

Acute graft‐versus‐host disease (aGvHD) remains a substantial cause of morbidity and mortality after allogeneic hematopoietic cell transplantation (allo‐HCT). Limited studies explore the association between specific major histocompatibility complex (MHC) alleles and aGvHD or transplant‐related mortality (TRM). After a noted clinical trend of higher TRM among a series of patients with aGvHD and MHC class I HLA‐
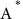
01:01, we retrospectively evaluated transplant outcomes of 404 adult allo‐HCT patients who underwent unmodified allografts between 03/2010 and 02/2017. HLA‐
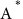
01:01 was expressed by 104 (25.7%) patients. In a univariate analysis, patients who underwent unmodified transplants and expressed HLA‐
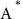
01:01 had a higher risk of TRM (HR 1.63 [95% CI: 1.05–2.53], *p* = 0.035). In a multivariate cause‐specific Cox model, HLA‐
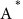
01:01 was significantly associated with TRM after adjusting for grade II–IV aGvHD, conditioning regimen intensity, and age at transplant (HR 1.59 (95% CI: 1.02–2.48) *p* = 0.039). There were no significant differences in overall survival (OS), aGvHD, or chronic GvHD based on HLA‐
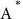
01:01 expression. With confirmation in a larger cohort, these findings have potential implications for the selection of allograft type, conditioning regimen, and post‐transplant monitoring for this high‐risk population.

## 1. Introduction

Acute graft‐versus‐host disease (aGvHD) remains a significant cause of morbidity and nonrelapse mortality after allogeneic hematopoietic cell transplantation (allo‐HCT) [1]. Several risk factors for the development of aGvHD have been identified including the degree of donor‐recipient HLA disparity, intensity of conditioning regimen, choice of GvHD prophylaxis, and graft source, among others [2]. These have led to the implementation of varied therapeutic strategies to decrease GvHD risk, including use of CD34+ selected/ex vivo T‐cell depleted (TCD) allografts, post‐transplant cyclophosphamide, and abatacept [3, 4].

The pivotal initial event in the development of GvHD is the recognition of antigens on host cells by donor‐infused immune cells [5]. Donor‐recipient HLA mismatch is an important risk factor associated with aGvHD. However, it may still develop even with fully HLA‐matched allele allografts [2, 6]. This may be explained by patient difference in minor histocompatibility antigens (miHAs) presented by HLA identical recipient alleles [2, 6, 7]. These miHAs are inherited independently from HLA between otherwise HLA‐identical siblings [8]. Though some major and minor histocompatibility complexes have been associated with increased or decreased risk of aGvHD in mismatched allo‐HCT [6, 7, 9], studies are limited in patients with matched alleles.

The skin is the organ most commonly affected by aGvHD [3, 10]. Cutaneous aGvHD is often the first clinical manifestation of the disease and typically demonstrates a high response to therapy [10, 11]. We identified a group of allo‐HCT patients in our clinic cohort who had severe refractory cutaneous aGvHD, however, all of whom expressed HLA‐
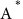
01:01. Although HLA‐A alleles have been implicated in a higher risk of aGvHD in mismatched transplants [12], the effect of HLA polymorphism on cutaneous aGvHD and transplant morbidity and mortality among HLA‐matched allo‐HCT patients remains an important unknown. Given our clinical observation of severe aGvHD among patients with this genotype, we hypothesized that HLA‐
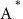
01:01 allele positivity is associated with more severe cutaneous aGvHD and worse allo‐HCT outcomes.

## 2. Methods

### 2.1. Patient and Graft Characteristics

This analysis included adult patients who underwent allo‐HCT at Memorial Sloan Kettering Cancer Center (MSK) between March of 2010 and February of 2017. HLA typing used high‐resolution, DNA sequence‐specific oligonucleotide typing for HLA‐A, ‐B, ‐C, ‐DRB1, and ‐DQB1 (5 allele level). Donor selection used matching at 10 HLA‐alleles. Typing for the HLA‐DPB1 allele was not routinely conducted and stored at our institution during the studied timeframe; thus, we were unable to assess matching at 12 HLA‐alleles. Patients received unmodified allografts. Cord blood transplant recipients, patients with donor‐recipient HLA‐match <10/10, TCD allografts, and second transplants were excluded. Patient demographics and clinical information about patient disease, treatments, and outcomes were obtained through retrospective review of the consensus data tracker maintained by the Adult Bone Marrow Transplant Service (BMT). Approval for this retrospective review was obtained from the MSKCC Institutional Review and Privacy Board and the need for informed consent was waived.

### 2.2. Conditioning Regimen, GVHD Prophylaxis, and Disease Risks

Conditioning regimens were classified as fully myeloablative or reduced intensity (RIC)/nonmyeloablative (NMA). Unmodified allo‐HCT recipients received calcineurin inhibitor‐based GvHD prophylaxis combined with methotrexate. Patients’ disease risk status was determined using the disease risk index (DRI) with clinical information derived from the BMT service consensus data tracker.

### 2.3. Study Definitions and Statistical Analysis

An institutional GvHD Consensus Committee established the diagnosis and staging of cutaneous aGvHD using clinical data as well as histological data when available. International Bone Marrow Transplant Registry criteria were used to finalize aGvHD grading [13]. The 2014 NIH Consensus Criteria were used for cGvHD grading [14]. For each patient with aGvHD and/or cGvHD, we assessed donor‐recipient HLA‐typing (including donor‐recipient relation and HLA‐
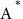
01:01 allele status), as well as time of onset, grade at onset, and highest grade of overall and cutaneous aGvHD.

Descriptive statistics like frequencies, medians, and ranges characterized the study cohort. All study endpoints were evaluated from the date of transplant. Causes of death were described according to the Copelan algorithm [15]. Transplant‐related mortality (TRM) was defined as deaths not caused by relapse or progression of disease. Cumulative incidence functions estimated the incidence of TRM and aGvHD to account for the competing events of non‐TRM vs relapse or death, respectively. Competing risks regression results were evaluated using the cause‐specific hazard approach. aGvHD covariates in the setting of competing risks regression for TRM were modeled as time‐dependent covariates. Differences in cumulative incidences of TRM and aGvHD were calculated using the Gray’s test. Survival estimates were compared between patients with and without the HLA‐
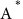
01:01 allele across the treatment groups using the log‐rank test. Statistical analyses were performed using R statistical software version 4.0.3.

## 3. Results

### 3.1. Patient and Graft Characteristics

We evaluated 404 patients. Table 1 summarizes the patient characteristics. Most patients (*n* = 282, 69.8%) received allo‐HCT after RIC or nonmyeloablative conditioning for the treatment of hematologic malignancies or high‐risk nonmalignant hematologic disorders. Patient age, gender, and race were similarly distributed across groups. Because all patients had 10/10 HLA‐allele matched donors, both donor and recipient either expressed HLA‐
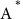
01:01 or did not. HLA‐
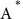
01:01 was expressed in 104 (25.7%) patients who had similar demographics to patients lacking HLA‐
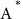
01:01 (Table 1). Donor‐recipient sex concordance differed between groups, with more male–male and fewer female‐male transplants in the HLA‐
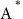
01:01‐expressing cohort. Two patients (0.5%) were homozygous for the HLA‐
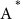
01:01 allele, while all other patients were heterozygous. Median follow‐up time in years was 4.47 (range: 0.22–8.48) for HLA‐
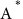
01:01 patients and 4.58 (range: 0.22–9.48) for patients without the allele.

**Table 1:** Patient demographics and clinical characteristics.

Characteristic	Unmodified, *N* = 404			
	Overall *N* = 404^a^	Non‐HLA‐ 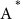 0101 *N* = 300^a^	HLA‐ 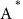 0101 *N* = 104^a^	*p*‐Value^b^
Sex				0.5
M	248 (61%)	181 (60%)	67 (64%)	
F	156 (39%)	119 (40%)	37 (36%)	
Age at HCT	57 (47, 66)	56 (47, 65)	58 (49, 67)	0.2
Race				0.7
Asian/Far East/Indian Subcontinent	20 (5.0%)	17 (5.7%)	3 (2.9%)	
Black/African American	18 (4.5%)	15 (5.0%)	3 (2.9%)	
Other	6 (1.5%)	5 (1.7%)	1 (1.0%)	
Pt refused to answer	16 (4.0%)	12 (4.0%)	4 (3.8%)	
White	344 (85%)	251 (84%)	93 (89%)	
Donor status				0.3
Related	160 (40%)	123 (41%)	37 (36%)	
Unrelated	244 (60%)	177 (59%)	67 (64%)	
Donor–recipient sex				0.089
F–F	66 (16%)	50 (17%)	16 (15%)	
F–M	81 (20%)	67 (22%)	14 (13%)	
M–F	90 (22%)	69 (23%)	21 (20%)	
M–M	167 (41%)	114 (38%)	53 (51%)	
GvHD prophylaxis				0.2
Bortezomib/methotrexate x4/tacrolimus	7 (1.8%)	4 (1.3%)	3 (2.9%)	
Cyclophosphamide + mycophenolate mofetil + sirolimus/tacrolimus	3 (0.8%)	3 (1.0%)	0 (0%)	
Cyclophosphamide ± sirolimus	5 (1.3%)	2 (0.7%)	3 (2.9%)	
Cyclosporine + methotrexate + sirolimus/tacrolimus/mycophenolate mofetil	6 (1.5%)	6 (2.0%)	0 (0%)	
Cyclosporine ± methotrexate	1 (0.3%)	0 (0%)	1 (1.0%)	
Maraviroc + methotrexate + tacrolimus	4 (1.0%)	2 (0.7%)	2 (2.0%)	
Methotrexate + mycophenolate mofetil + sirolimus/tacrolimus ± solumedrol	22 (5.5%)	18 (6.1%)	4 (3.9%)	
Methotrexate + sirolimus/tacrolimus	339 (85%)	253 (85%)	86 (84%)	
Mycophenolate mofetil + sirolimus/tacrolimus	8 (2.0%)	6 (2.0%)	2 (2.0%)	
Tacrolimus ± sirolimus	4 (1.0%)	3 (1.0%)	1 (1.0%)	
Unknown	5	3	2	
Disease				0.8
Aplastic anemia	2 (0.5%)	2 (0.7%)	0 (0%)	
Hodgkin’s disease	20 (5.0%)	16 (5.3%)	4 (3.8%)	
Leukemia	194 (48%)	145 (48%)	49 (47%)	
Leukemia (second primary)	1 (0.2%)	1 (0.3%)	0 (0%)	
Multiple myeloma	2 (0.5%)	1 (0.3%)	1 (1.0%)	
Myelodysplastic syndrome	47 (12%)	31 (10%)	16 (15%)	
Myeloproliferative disorder	10 (2.5%)	8 (2.7%)	2 (1.9%)	
Myeloproliferative disorder (second primary)	2 (0.5%)	2 (0.7%)	0 (0%)	
Non‐Hodgkin’s lymphoma	107 (26%)	77 (26%)	30 (29%)	
Non‐Hodgkin’s lymphoma (second primary)	14 (3.5%)	12 (4.0%)	2 (1.9%)	
Nonmalignant Hemat disorders	5 (1.2%)	5 (1.7%)	0 (0%)	
Risk				0.8
High	99 (25%)	73 (25%)	26 (25%)	
Intermediate	144 (36%)	110 (37%)	34 (33%)	
Low	136 (34%)	97 (33%)	39 (38%)	
Not applicable	22 (5.5%)	17 (5.7%)	5 (4.8%)	
Unknown	3	3	0	
Source				0.5
Allo BM unmodified	45 (11%)	31 (10%)	14 (13%)	
Allo PBSC and BM unmodified	1 (0.2%)	1 (0.3%)	0 (0%)	
Allo PBSC unmodified only	358 (89%)	268 (89%)	90 (87%)	
Intensity: ablative vs. reduced/non‐ablative				0.6
Reduced/non‐ablative	282 (70%)	207 (69%)	75 (72%)	
Ablative	122 (30%)	93 (31%)	29 (28%)	
Donor CMV atatus				0.8
Inconclusive	1 (0.2%)	1 (0.3%)	0 (0%)	
Negative	208 (52%)	151 (51%)	57 (55%)	
Not done	1 (0.2%)	1 (0.3%)	0 (0%)	
Positive	192 (48%)	145 (49%)	47 (45%)	
Unknown	2	2	0	

^a^ *n* (%); median (Q1, Q3).

^b^Pearson’s Chi‐squared test; Wilcoxon rank sum test; and Fisher’s exact test.

### 3.2. TRM

**Figure 1:**
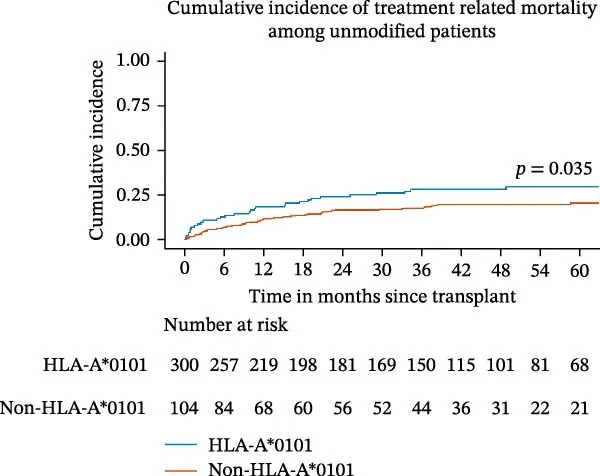
Transplant‐related mortality among unmodified grafts.

In a univariate analysis, patients who expressed HLA‐
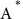
01:01 had a significantly higher risk of overall TRM than nonexpressors (HR 1.63 [95% CI: 1.05–2.53], *p* = 0.035) (Figure 1). Age at transplant, RIC/NMA versus ablative conditioning regimen intensity, and grade II–IV overall aGvHD were also significantly associated with an increased risk of TRM. The disease type was not associated with TRM. No significant difference was seen between HLA‐
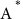
01:01 expressors and nonexpressors among strictly unrelated donors or related donors. Upon multivariate analysis, an increase in risk of TRM for HLA‐
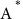
01:01 patients remained significant [HR 1.59 (95% CI: 1.02–2.48) *p* = 0.039] after adjusting for age at transplant, ablative versus RIC/NMA conditioning regimen intensity, and grade II–IV aGvHD. Those who developed grade II–IV aGvHD also had an increased risk of TRM [HR 1.71 (95% CI: 1.08–2.72) *p* = 0.022) in the multivariate setting (Table 2).

**Table 2:** Multivariate analysis for treatment related mortality.

Characteristic	HR	95% CI	*p*‐Value
Overall aGVHD II–IV	1.71	1.08, 2.72	**0.022**
HLA‐ 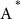 0101 status			
Non‐HLA‐ 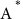 0101	—	—	**0.039**
HLA‐ 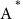 0101	1.59	1.02, 2.48	
Intensity: ablative vs. reduced/non‐ablative			
Reduced/non‐ablative	—	—	—
Ablative	0.60	0.36, 1.02	0.058
Age at HCT	1.01	1.00, 1.03	0.15

Abbreviations: CI, confidence interval; HR, hazard ratio.

*Note*: Bold values indicate statistical significance.

### 3.3. Overall Survival (OS)

In the univariate setting, there was a significant association between HLA‐
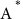
01:01 expression and OS [HR 1.63 (95% CI:1.05−2.53), *p* = 0.035]. The incidence of both stage 3–4 cutaneous aGvHD [HR 6.35 (95% CI: 4.70–8.58, *p* < 0.001] and overall grade III–IV aGvHD [HR 7.32 (95% CI: 5.36, 10.0), *p* < 0.001] were significantly associated with OS. No significant difference was seen between HLA‐
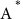
01:01 expressors and nonexpressors among strictly unrelated donors or related donors. (Table 3).

**Table 3:** Univariate analysis of study endpoints.

Estimate	HR (95% CI) for HLA‐ 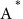 01:01 present vs. absent	*p*‐Value
TRM	1.63 (1.05, 2.53)	**0.035**
OS	1.30 (0.95, 1.76)	0.10
Stage 2–4 cutaneous aGvHD	1.32 (0.81, 2.15)	0.26
Grade II–IV aGvHD	0.95 (0.69, 1.33)	0.79

*Note*: Bold values indicate statistical significance.

### 3.4. Causes of Death

Table 4 lists the causes of death for the 199 patients who died. TRM was the cause of death for 46% of the deceased patients (*n* = 92). The most common causes of death were disease progression (*n* = 107, 54%), GvHD (*n* = 50, 25%), or infection (*n* = 20, 10%). No significant difference in cause of death was seen based on HLA‐
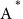
01:01 expression (*p* = 0.3).

**Table 4:** Patient causes of death.

Cause of death	Overall, *N* = 199^a^		Non‐HLA‐ 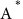 01:01, *N* = 141^a^		HLA‐ 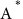 01:01, *N* = 58^a^
Disease progression	107 (53.8%)		80 (56.7%)		27 (46.6%)
GvHD	50 (25.1%)		35 (24.8%)		15 (25.9%)
Infection	20 (10.1%)		11 (7.8%)		9 (15.5%)
Organ failure	13 (6.5%)		7 (5.0%)		6 (10.3%)
Secondary nonhematologic malignancy	3 (1.5%)		3 (2.1%)		0 (0.0%)
Toxicity from treatment	3 (1.5%)		2 (1.4%)		1 (1.7%)
Other/unknown	3 (1.5%)		3 (2.1%)		0 (0.0%)

^a^ *n* (%).

### 3.5. Incidence of Cutaneous and Overall aGVHD

**Figure 2:**
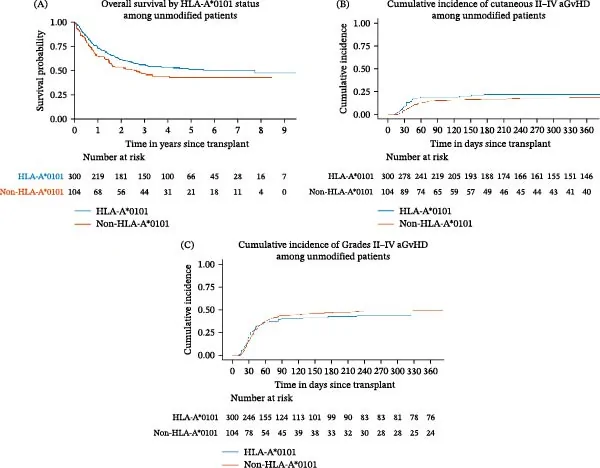
Overall survival (A), stage 2–4 cutaneous aGvHD (B), and Grade II–IV aGvHD (C) among unmodified grafts.

At day 365 post‐HCT, patients expressing HLA‐
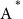
01:01 had a similar incidence of stage 2–4 cutaneous aGvHD (22% vs. 19%, *p* = 0.37; HR 1.32 [0.81, 2.15], *p* = 0.26) and cumulative incidence of grade II–IV aGvHD (45% vs. 50%, *p* = 0.60; HR 0.95 [0.69, 1.33], *p* = 0.79). The results are shown in Table 3 and Figure 2. In univariate analyses, patients with unrelated donors had a significantly increased risk of grade II–IV overall aGvHD compared to those with related donors (HR = 1.79 [95% CI: 1.32–2.43], *p* < 0.001).

### 3.6. Incidence of cGvHD

Chronic GvHD data was available for 177 patients. Of these, 15% (*n* = 27) had any reported cGvHD. No difference in cGvHD incidence at day 365 was seen between patients with and without HLA‐
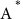
01:01 expression (15% vs. 15%).

## 4. Discussion

Though we first clinically noted the HLA‐
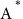
01:01 allele in our population as potentially associated with more severe cutaneous aGvHD leading to increased mortality, our analyses did not show a difference in cutaneous GvHD severity; however, they did detect increased TRM in unmodified recipients who expressed this allele or who developed grade II–IV aGvHD within our cohort. Given the high morbidity and mortality associated with GvHD, evaluations of prophylactic and risk‐reducing measures remain of great interest. TRM remains relevant after allo‐HCT, with incidence ~20%–40% [16]. While HLA mismatch between donor and recipient is the most well‐established risk factor for the development of aGvHD and TRM after allo‐HCT [6], investigations into the possible link between the expression of a specific HLA polymorphism and this risk in matched patients is limited.

HLA‐
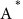
01:01 is a common allele in populations worldwide, with up to 28.7% of individuals expressing the allele in some United States populations [17]. HLA‐
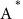
01:01 expression also correlates with autoimmune disease, but has not previously been associated with allogeneic transplantation outcomes or GvHD [18–20]. Several HLA alleles have been associated with the risk of aGvHD or an effect on TRM (Table S1). Prior studies have found that among patients with mostly hematologic diseases, various HLA alleles may confer increased risk for or protection from development of aGvHD, including HLA‐
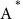
10, 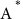
26, and a number of HLA‐B alleles [21–31]. Only three studies have reported matched alleles’ impact on TRM, and among these, only HLA‐
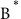
07 was associated with increased TRM (*p* = 0.04) [25, 27, 29]. These studies’ varied findings, in which all known alleles of certain major histocompatibility complex (MHC) molecules were evaluated, underscore the difficulty in determining HLA‐based risk factors of aGvHD in matched patients. Furthermore, these studies did not address differences in cutaneous versus overall aGvHD, and most did not analyze the effect on TRM.

The implications of a higher risk of TRM among patients with this allele include a potential re‐examination of donor selection in allo‐HCT. If HLA allele‐matched patients still experience poor outcomes, minor histocompatibility matching may further reduce this risk. Autosomal miHA may develop and subsequently be inherited through several mechanisms, including via single nucleotide polymorphisms or frameshift deletions in primary gene transcripts. HLA‐
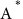
01:01‐restricted miHA disparities associated with higher rates of aGvHD and decreased survival among MHC‐matched allo‐HCT patients have been identified [32–38]. Notably the Y‐chromosome‐specific HY antigen A1‐HY, which is restricted by 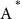
0101, has been shown to induce strong T‐cell recognition compared to its X‐chromosome counterpart [37]. This immune response suggests that allelic variation in miHA may be key drivers in the development of GHvD. However, further studies have failed to reveal an association between mismatch of most minor antigens and the incidence of aGvHD or TRM [39, 40]. Future investigations may elucidate the impact of matched miHAs on allo‐HCT outcomes and their role in the balance between aGvHD and the graft‐versus‐leukemia effect [41].

Our findings of an increased risk of TRM among unmodified allograft recipients suggest that particular attention to graft conditioning is further warranted. The TCD graft may be preferred, and RIC/NMA conditioning intensity may also help improve outcomes in these patients [42, 43]. Further investigations may be warranted to determine the optimal regimen to reduce TRM.

Our results are limited by this study’s retrospective design and number of patients. Additionally, cGvHD consensus data was not collected until 2015, limiting our analyses of cGvHD outcomes. HLA‐DPB1 allele data are not stored in the database at our institution, and we are unable to assess the matching status of this locus. Infection parameters such as CMV and EBV serostatus were also not recorded for the majority of patients and were thus unable to be included in our analyses. These factors, along with linkage disequilibrium patterns, could be unaccounted contributors to increased TRM in the study cohort. To account for confounding variables, multivariate analyses were conducted. Although assessing HLA‐
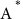
01:01 in isolation does not allow for examination of patterns of linkage disequilibrium in this region, we found no significant differences between HLA‐
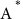
01:01 expressors and nonexpressors among unrelated or related donors. Notably, the prevalence of the HLA‐
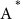
01:01 allele in our population (25.7% of our patient cohort) was similar to that of the Caucasian population in the United States, supporting the generalizability of our findings to patients of similar ethnic origin. Though our cohort is small, our observations may serve as a point from which to further study the association between specific HLA alleles and transplant related outcomes.

## 5. Conclusions

In summary, HLA‐
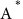
01:01 expression was found to be an independent risk factor for TRM in our cohort of unmodified allo‐HCT recipients, even after 10/10 HLA‐matched transplantation. This finding may have clinical implications, and these results should be confirmed in a larger multicenter population before considering changes in clinical practice with respect to the allograft type, conditioning regimen, and post‐transplant monitoring.

## Funding

This study was supported in part by the NIH/NCI Cancer Center Support Grant P30‐CA008748.

## Conflicts of Interest

James W. Young owns common stock in Amgen, Merck, and Pfizer. Sergio Giralt receives research funding from Miltenyi Biotec, Takeda Pharmaceutical Co., Celgene Corp., Amgen Inc., Sanofi, Johnson and Johnson, Inc., and Actinium Pharmaceuticals, Inc.; and is on the Advisory Boards for Kite Pharmaceuticals, Inc., Celgene Corp., Sanofi, Novartis, Johnson and Johnson, Inc., Amgen Inc., Takeda Pharmaceutical Co., Jazz Pharmaceuticals, Inc., and Actinium Pharmaceuticals, Inc. Doris M. Ponce receives research funding from Incyte and has served as an advisory board member for Evive Biotechnology (Shanghai) Ltd (formerly Generon [Shanghai] Corporation Ltd), Kadmon Corporation, CareDx, Incyte, and Ceramedix. Ann A. Jakubowski owns common stock in Novartis. Alina Markova receives research funding from Incyte Corporation, Kintara, Novartis, and Amryt Pharma; consults for ADC Therapeutics, AstraZeneca, Blueprint Medicines, Alira Health, OnQuality, Protagonist Therapeutics, Sanofi Genzyme, and Janssen; and receives royalties from UpToDate. Emily A. Cowen, Samantha Brown, Rachel E. Reingold, Sarah Samorodnitsky, Maira Fonseca, Joseph R. Stoll, Sean M. Devlin, Samira A. Fatmi, and Molly Maloy declare no conflicts of interest.

## Supporting Information

Additional supporting information can be found online in the Supporting Information section.

## Supporting information


**Supporting Information** Table S1: Classical MHC molecule associations with aGvHD and TRM.

## Data Availability

The data that support the findings of this study are available from the corresponding author upon reasonable request.
