# Supplementary material for: HLA‐A ∗01:01 Allele Is Associated With Increased Risk of Transplant‐Related Mortality After MHC Matched Unmodified Allogeneic Hematopoietic Stem Cell Transplantation
Source: J Immunol Res. 2026 Aug 31;2026:7237488. doi: 10.1155/jimr/7237488 (PMC13528444; doi:10.1155/jimr/7237488)
Supplement: Supplementary file 1 [file JIMR-2026-7237488-s001.docx]

| **Supplemental Table 1: Classical MHC molecule associations with aGvHD and TRM** | | | | | | | | |
| --- | --- | --- | --- | --- | --- | --- | --- | --- |
| **Study** | **Patients (N)** | **Patient characteristics** | **Conditioning regimen (n)** | **GvHD prophylaxis (n)** | **Alleles examined** | **Associations with aGvHD (grade II-IV)** | **Associations with TRM** | |
| Storb 1983 | 130 | Allo-HCT HLA-identical sibling donors SAA patients (130) | High-dose Cy (130) | MTX | HLA-A and B | HLA-B18: increased risk (RR 3.57, *p*=0.01) HLA-B8: decreased risk (RR 0.47, *p*=0.05) | Not examined | |
| Bross 1984 | 136 | Allo-HCT  HLA-identical sibling donors  Aplastic anemia, acute or chronic leukemia, or other hematologic malignancy | Cy  Pro + ATG + Cy  Pro + ATG + TBI  Bu + Cy  Cy + TBI  Cy + Fractional TBI | MTX  Cy | HLA-A, B, and C | HLA-Cw4, B49, B50: increased risk (*p*<0.05)  HLA-Aw19: decreased risk (*p*<0.01) | Not examined | |
| Weisdorf 1991 | 469 | Allo-HCT HLA-identical sibling donors Most had hematological malignancy | TBI + Cy High-dose cytarabine Other TLI + Cy Bu + Cy | MTX + ATG + prednisone (263) MTX (102) TCD (56) Other (47) None (1) | HLA-A, B, and DR | In patients with known DR typing: HLA-A26: increased (RR 2.72 [95% CI: 1.37-5.41], *p*=0.012) DR allele (not DR3): increased (RR 2.05 [05% CI: 1.17-3.61], *p*=0.006)  In whole population studied: HLA-A26: increased (RR 2.06 [95% CI: 1.28-3.33], *p*=0.007)  DR3: decreased (*p*=0.01) | Not examined | |
| Smyth 1993 | 51 | Allo-HCT Mostly HLA-identical related donors, 3 unrelated Various diseases | Not reported | MTX (13)  CsA + MTX (33)  TCD (8) | HLA-A, B, and DR | HLA-B7: decreased risk (p=0.005) HLA-B44: increased risk (p=0.02) | Not examined | |
| Clark 2001 | 751 | Allo-HCT HLA-identical related donors CML patients | Not reported | CsA +/- MTX (489) TCD and other (200) Misc (62) | HLA-A, B, and DR | HLA-A3: increased risk (RR 1.52 [95% CI: 1.02-2.28], *p*=0.03) HLA-DR1: decreased risk (RR 0.59 [95% CI: 0.35-0.98], *p*=0.03) | No significant difference found in TRM for alleles examined | |
| Remberger 2002 | 493 | Allo-HCT HLA-identical sibling donors Most had hematological malignancy | Cy (32) TLI + Cy (7) TBI + Cy (306) Fractionated TBI + Cy (23) Bu + Cy (106) Fludarabine + Cy (7) Fludarabine + Bu (2) Fludarabine + TBI (9) | MTX (67) CsA (51) MTX + CsA (323) TCD (28) Prednisolone + CsA (3) CsA + MMF (21) | HLA-A and B (serologic) HLA-DR (genomic) | HLA-A10: increased risk (OR 1.87 [95% CI: 1.01-3.59], *p*=0.04) HLA-B7: increased risk (OR 1.72 [95% CI: 1.06-2.78], *p*=0.03) | HLA-B7: higher TRM (*p*=0.04) | |
| Stern 2006 | 192 | Allo-HCT HLA-identical sibling donors Acute or chronic leukemia, or non-Hodgkin lymphoma patients | TBI + Cy (23) TBI + Cy + VP16 (112) Bu + Cy (8) Other (16) TBI + Flu (33) | CsA (39) CsA + MTX (120) CsA + MMF (33) | HLA-DRB1 (molecular) for DR15 | aGvHD was less severe and less frequent in DR15 patients, but findings were not significant | No significant difference found in TRM in patients with DR15 | |
| Davidson 2007 | 286 | Allo-HCT HLA-identical sibling donors | Not reported | Not reported | HLA-A, B, C, DRB1, and DQB1 | Not examined | Not examined, but improved OS in patients with DR15 (*p*=0.0143) and improved OS in DR15 positive patients with aGvHD (*p*=0.0196) | |
|  | 133 | Allo-HCT HLA-identical unrelated donors | Not reported | Not reported | HLA-A, B, C, DRB1, and DQB1 | Not examined | | Not examined, but in 88 adult patients, DR15 associated with decreased OS (*p*=0.0246), and in adult patients with aGvHD, DR15 was associated with decreased OS (*p*=0.0986) No association seen in pediatric patients. |
| Cardozo 2012 | 176 | Allo-HCT HLA-identical sibling donors Most had hematological malignancy | Cy  Bu + Cy  Bu + Cy + other  Cy + TBI^46,47^ | CsA + MTX  CSP + Prednisone^46,47^ | HLA-A and B (serologic) HLA-DRB1 and DQB1 (molecular) | HLA-A10: positive association (p=0.00070) HLA-A26: positive association (p=0.002) HLA-B55: positive association (p=0.001) HLA-DRB1*15: positive association (p=0.0211) HLA-DQB1*05: positive association (p=0.038) | Not examined | |
| Battiwalla 2012a | 2891 | Allo-HCT HLA-identical sibling donors AML, ALL, MDS, or CLL patients | TBI + Cy +/- other (991) TBI +/- other, no Cy (216) Cy + Bu +/- other, no TBI (1684) | CsA + MTX +/- other (2891) | HLA-DRB1 (molecular) for DR15 | No significant difference in incidence of aGvHD with DR15 | No significant difference in TRM with DR15 | |
| Battiwalla 2012b | 1204 | Allo-HCT HLA-identical sibling donors SAA patients | Cy + ATG (935) Cy (182) Cy + limited field irradiation +/- ATG (34) Bu + Cy +/- ATG (178) Flu + Cy +/- ATG (38) Flu + Bu +/- ATG (24) Flu + low dose TBI/limited field irradiation +/- other (3) Other (10) | CsA + MTX + other (1045) CsA +/- other (122) Tacrolimus +/- other (29) Tacrolimus (8) | HLA-DRB1 (molecular) for DR15 | No significant difference in incidence of aGvHD with DR15 | No significant difference in TRM with DR15 | |
| Abbreviations: Cyclophosphamide (Cy), Total lymphoid irradiation (TLI), Total body irradiation (TBI), Busulphan (Bu), Methotrexate (MTX), Cyclosporin (CsA), T-cell depletion (TCD), Mycofenolate mofetil (MMF), ATG (antithymocyte globulin), Pro (procarbazine), Severe Aplastic Anemia (SAA), Chronic Myelogenous Leukemia (CML), Acute Myelogenous Leukemia (AML), Acute Lympblastic Leukemia (ALL), Myelodysplastic Syndrome (MDS), Chronic Lymphocytic Leukemia (CLL) | | | | | | | | |
